# Characteristics of Patients That Do Not Initially Respond to Intravenous Antihypertensives in the Emergency Department: Subanalysis of the CLUE Trial

**DOI:** 10.5811/westjem.2015.1.23308

**Published:** 2015-03-17

**Authors:** Caroline E. Freiermuth, Abhinav Chandra, W. Frank Peacock, Site Investigators

**Affiliations:** * Duke University Medical Center, Division of Emergency Medicine Durham, North Carolina; † Kaiser Permanente, South Sacramento, Sacramento, California; ‡ Baylor College of Medicine, Department of Emergency Medicine, Houston, Texas

## Abstract

**Introduction:**

Hypertensive emergency has a high mortality risk and the treatment goal is to quickly lower blood pressure with intravenous (IV) medications. Characteristics that are associated with non-response to IV antihypertensives have not been identified. The objective is to identify patient characteristics associated with resistance to IV antihypertensives.

**Methods:**

This was a subanalysis of patients enrolled in the previously described comparative effectiveness trial of IV nicardipine vs. labetalol use in the emergency department (CLUE) study, a randomized trial of nicardipine vs. labetalol. Non-responders were defined as those patients who did not achieve target systolic blood pressure (SBP), as set by the treating physician, within thirty minutes of IV antihypertensive medication, +/− 20mmHg. Stepwise logistic regression was used to identify covariates associated with the measurement outcomes.

**Results:**

CLUE enrolled 226 patients, 52.7% female, 76.4% black, mean age of 52.6±14.6 years, of whom 110 were treated with nicardipine and 116 with labetalol. The median (IQR) initial systolic blood pressure was 211mmHg (198, 226), 210 (200, 230), and 211mmHg (198, 226), for the total, non-responder, and responder cohorts, respectively (p-value=0.65, 95% CI [−5.8–11.3]). Twenty-nine were non-responders, 9 in the nicardipine and 20 in the labetalol group. In univariate analysis, several symptoms suggestive of end organ damage were associated with non-response. After multiple variable logistic regression (AUC = 0.72), treatment with labetalol (OR 2.7, 95% CI [1.1–6.7]), history of stroke (OR 5.4, 95% CI [1.6–18.5]), and being male (OR 3.3, 95% CI [1.4–8.1]) were associated with failure to achieve target blood pressure.

**Conclusion:**

Male gender and history of previous stroke are associated with difficult to control blood pressure.

## INTRODUCTION

Hypertension, defined as a blood pressure of greater than 140/90mmHg, affects almost one fourth of the adult U.S. population.^1^ Complications from hypertension include stroke, cardiovascular disease, and renal failure, among others. It has been estimated that 7.1 million deaths worldwide can be attributed to hypertension and its long term effects.^2^ Suboptimal blood pressure control is also thought to be responsible for up to 62% of cerebrovascular disease and 49% of ischemic heart disease.^2^

The frequency of hospitalizations for a hypertensive emergency increased from 101/100,000 U.S. adults in 2000 to 111/100,000 U.S. adults in 2007, an average increase of about 1.1% over the seven year time period.^3^ It is recommended that any patient presenting with hypertensive emergency, defined as a blood pressure greater than 180/120 in conjunction with evidence of end organ damage,^4^ should be given intravenous (IV) medications to immediately lower blood pressure, as the one year mortality rate for untreated hypertensive emergency is as high as 90%.^5^ There is no clear evidence as to the optimal pharmacological approach to blood pressure control in hypertensive emergency. A recent Cochrane review concluded that there is wide overlap in blood pressure lowering between agents, and that therefore it is difficult to recommend any particular antihypertensive for treatment of hypertensive emergencies.^6^ Characteristics that may predispose patients to having resistant hypertension have been reported for oral medication therapy, but none have been defined for IV therapy.^7^

It has long been recognized that overly aggressive lowering of blood pressure can be harmful, and even catastrophic in some cases. Therefore, *a priori* identification of patients that may be resistant to initial treatments would be helpful to identify those whom would benefit from a more aggressive approach, such as more rapid titration or early addition of a second agent. Identifying patients who may need prolonged IV antihypertensive therapy is important because it allows for appropriate disposition and monitoring of the patient. Patients receiving prolonged IV antihypertensive medications require close clinical monitoring to avoid overshooting target blood pressure and other significant hemodynamic consequences. The objective of this analysis is to identify characteristics of those patients resistant to parenteral antihypertensives for the treatment of hypertensive crisis in the emergency department (ED).

## METHODS

### Study Design

This study is a descriptive sub-analysis of the multicenter Evaluation of IV Cardene (Nicardipine) and labetalol use in the emergency department (CLUE) trial, a U.S.-based, prospective, randomized, open-label study of the management and outcomes for patients with acute severe hypertension treated with IV antihypertensive therapy in the emergency department.^8^ The study was approved by the institutional review boards at all sites. The methods for the primary CLUE trial were previously published and described in detail elsewhere^8^ and registered at ClinicalTrials.gov with identifier NCT00765648.

### Study Setting and Population

Adult patients (aged ≥18 yrs) who presented to one of 13 participating hospitals’ emergency departments with acute severe hypertension were eligible for inclusion. Patients provided written informed consent, including authorization to use protected health information, for inclusion into the study. Acute severe hypertension was defined as two consecutive systolic blood pressure readings, at least ten minutes apart, of 180mmHg or greater.

Patients were ineligible if they had specific contraindications to receiving either a beta blocker or a calcium channel blocker. Patients were also excluded if they met any of the following criteria: use of any investigational drug within 30 days, pregnant or breast-feeding, contraindications or allergy to beta-blockers and calcium channel blockers (per food and drug administration [FDA]-approved labeling for nicardipine and labetalol), advanced aortic stenosis, bronchial asthma, overt cardiac failure, greater than first-degree heart block, cardiogenic shock, severe bradycardia, obstructive airway disease, decompensated heart failure or a known left ventricular ejection fraction <35%, history of stroke within 30 days, known impaired hepatic function, suspected myocardial infarction (MI), suspected aortic dissection, suspected cocaine use as the cause of ED presentation, or if they were concurrently receiving any IV antihypertensive medication.

### Protocol

After obtaining informed written consent, the treating physician was asked to define a target systolic blood pressure with a range of ± 20mmHg prior to patients being randomized to IV labetalol or nicardipine per set protocol.^8^ Medications were initiated within 30 minutes after randomization. FDA recommendations regarding dosing of the drugs used in this study were provided to the treating physicians, and the physician determined the dosing and the frequency of titration for each drug. Labetalol was administered as bolus doses at varying amounts and nicardepine was administered as a continuous infusion, titrated as needed. Descriptive, historical, and investigational clinical data were collected as soon as possible after enrollment, but this step was not required prior to the initiation of study drug. Data collected included past medical history and available laboratory data. Medications taken one week prior to screening, and the assessment of baseline signs and symptoms, were documented. At discharge, adverse events were recorded. Intensive care unit or hospital stay, date and cause of any deaths (with autopsy data if available), were also recorded.

### Measurements

Blood pressure recordings were taken with an automatic cuff every five minutes during the 30 minutes following initiation of treatment. Vital signs and adverse events were also monitored for six hours after initiation of IV antihypertensive or until discharge from the ED, whichever came first. The presence of end organ damage was also recorded. End organ damage was defined as having any one of the following symptoms suggestive of a hypertensive emergency at time of presentation: chest pain, shortness of breath, epigastric discomfort, syncope, dizziness, blurred vision, diplopia, diminished level of consciousness, confusion, hematuria, or development of acute ischemic changes on a twelve lead electrocardiogram. Although not all of these symptoms are included in the traditional definition for hypertensive emergency, the study group wanted to ensure that it captured patients with atypical symptoms for acute coronary syndrome. For this subanalysis, patients who met the target blood pressure (BP) within 30 minutes were defined as responders and those who did not meet target BP were defined as non-responders.

### Data Analysis

Categorical variables were compared by using Chi-square or Fisher’s Exact test, and continuous variables by Student’s T-test or Wilcoxon rank-sum test if the variable was not normally distributed. A multivariable logistic model to assess the risk factors for non-responders within 30 minutes, after controlling for site differences, was developed. Missing values were not imputed and only observed values were used for the multivariable analyses. All baseline variables with no more than 10% missing data points were considered for inclusion into the adjusted model. A stepwise elimination procedure was used to determine the final model. Risk factors with univariate p-value ≤0.10 were considered and included in the stepwise elimination procedure. All variables with a p-value <0.05 were included in the final model. The final multivariable logistic model included the variables of treatment drug, gender and no history of stroke. All statistical analyses were performed using SAS version 9.2 (SAS Institute, Cary, NC).

## RESULTS

**Figure 1:**
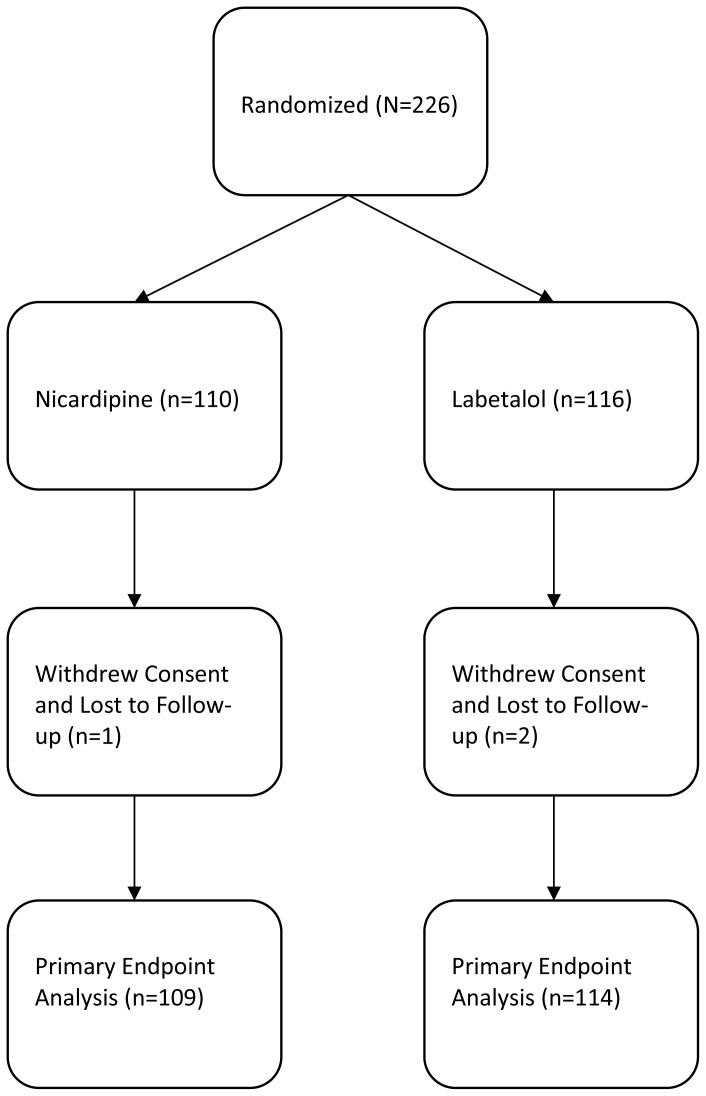
Flow diagram for CLUE enrollment.

A total of 226 patients were enrolled in the initial trial, 109 (49%) in the nicardipine group and 114 (51%) in the labetalol group (Figure 1). One patient in the nicardipine group withdrew and two patients from the labetalol group withdrew from the study, so 223 patients were included in the final endpoint analysis. Twenty-nine (13%) patients, 9 in the nicardipine group and 20 in the labetalol group, did not meet their target systolic blood pressure target. The overall population (n=223) had a mean age of 52.4 years, 105 (47.1%) were males, and 171 (77%) were African American (Table 1). Age, race and gender were similar between the responder and non-responder groups (Table 1). Characteristics comparing the nicardepine and labetalol groups were reported in Table 2 in the parent paper. Patients in the nicardepine group were more likely to be diabetic or have hyperlipidemia and patients in the labetalol group were more likely to have a social history of past or current smoking.

Presenting vital signs were similar throughout all subpopulations. The median (IQR) initial systolic blood pressure was 211mmHg (198, 206), 210 (200, 230), and 211mmHg (198,226), for the total, non-responder, and responder cohorts. Further, the minimum to maximum range of presenting systolic blood pressure was 163 to 275, 184 to 264, and 163 to 275mmHg for the total, non-responder, and responder populations. Finally, the mean initial presenting heart rate varied by approximately 1 beats per minute (bpm) for all cohorts (86±17, 85±16, and 86±17bpm, for the total, non-responder, and responder groups).

Non-responders were more likely to be male. They were also more likely to have previous medical history of stroke. Further, being a non-responder was associated with an altered level of consciousness, epigastric discomfort or palpitations at presentation. Non-responders were less likely to be taking antiadrenergic medications (Table 2). An elevated serum creatinine at presentation was associated with non-response to IV antihypertensive therapy. However, only ¼ of the non-responders were dialysis dependent, and there was no difference in proportion of responders vs non-responders who were dialysis dependent (Table 1).

As expected, non-responders spent less time in the pre-specified target BP range than responders. In fact, non-responders clearly represented a cohort of extremely resistant hypertensive patients as none were noted to have a BP reading within the target range during the entire thirty minute period. This compared to responders who had a median of 4 out of 6 BP readings within the target range (Table 3). Only one-third of the non-responders fell within 5mmHg of target systolic blood pressure (Table 4).

**Figure 2:**
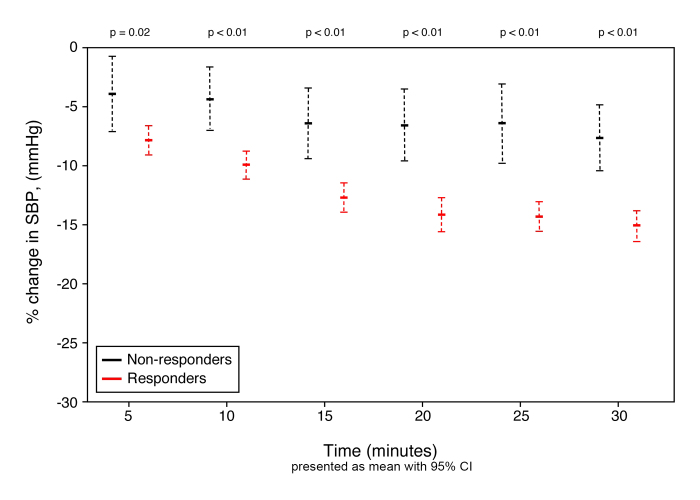
Change in systolic blood pressure measurements over time. *SBP*, systolic blood pressure

Although responders and non-responders were noted to have medication titrated the same amount of times within the thirty minute study period, responders received less drug overall due to smaller doses required to achieve blood pressure control within 30 minutes. Despite higher overall doses, non-responders had a significantly lower percent change in systolic blood pressure when compared to responders (Figure 2). With regard to adverse events, there was no statistical difference between responders and non-responders, including bradycardic episodes, defined as a heart rate below 60bpm (Table 3).

After adjusting for significant baseline variables by multivariate logistic regression, including forcing the study site into the model, randomization to treatment with labetalol (OR 2.7, p-value=0.028, 95% CI [1.1–6.7]), having a past medical history of stroke (OR 5.4, p-value=0.008, 95% CI [1.6–18.5]), or being male (OR 3.3, p-value=0.008, 95% CI [1.4–8.1]) were independently associated with failure to achieve a systolic blood pressure in the target range within 30 minutes of the start of IV anti-hypertension therapy (C statistic for the model = 0.72, Pearson’s goodness of fit test p-value=0.76).

## DISCUSSION

The primary goal of the CLUE study was to determine whether there was a difference in achieving target blood pressure within thirty minutes using nicardipine vs. labetalol. To the best of our knowledge, patient characteristics that are associated with resistance to parenteral antihypertensives in the ED have not been previously described. If patients resistant to BP control can be identified at presentation, more aggressive ED therapy may prevent further complications. Early ED identification of patients who will predictably have a poor response to BP control interventions may also assist emergency physicians in making appropriate inpatient dispositions decisions. Our univariate analysis suggests that presenting with select symptoms suggestive of end organ damage, including altered level of consciousness, epigastric pain and palpitations, increases the likelihood that blood pressure will not be lowered to target level within thirty minutes of treatment initiation. Our multivariate analysis indicates that being male and having history of a previous stroke also increases that likelihood.

The overall prevalence of hypertension is relatively similar between men and women, although men have a higher prevalence below the age of 60, and women have the higher prevalence after age 60.^1^ According to a recent analysis of the National Health and Nutrition Examination Survey (NHANES), women were more likely to be receiving medication for hypertension, but were less likely to have their blood pressure adequately controlled.^9^ In this analysis of the CLUE trial, there were no differences between responders and non-responders with regards to prior antihypertensive treatment. Although NHANES revealed that women were more resistant to oral antihypertensives, we found them to be less resistant than males to IV antihypertensives in the ED.

Hayes and Taler reviewed the literature regarding gender differences in chronic HTN to determine if treatment options for women should be different than those for men.^10^ Factors they considered to play a possible role in increased blood pressure in women included use of oral contraceptives and renal artery stenosis, which has a female predominance. It has been postulated that estrogen may be protective against end organ damage from hypertension,^11^ although Reckelhoo^12^ emphasized the role of the renin-angiotensin system (RAS) in blood pressure regulation. Studies on rats reveal that estrogen does not seem to affect the rates of hypertension, but that the lack of androgens is the key difference between males and females in blood pressure regulation.^12^ Men are known to have higher levels of renin than females.^13^ This may explain some of the difficulty in controlling blood pressure, since the CLUE trial used medications that did not target the RAS. Another study revealed that patients with difficult to control blood pressure, defined as lack of adequate response despite three oral agents, were found to have higher aldosterone levels. This elevation was most significant in men, despite correction for menopausal status of women.^14^

There are multiple physiologic differences between males and females that may contribute to development of hypertension, many of which disappear after menopause. These differences include higher cardiac index, higher HR and lower peripheral resistance and blood volume.^15^ Males are also known to have greater left ventricular mass, despite correction for weight, height, body mass index, and inotropic state.^16^ All of these factors may lead to differences in response to antihypertensives. Menopause status was not a recorded variable in this study, but the mean age of 52 suggests that many female patients were likely pre-menopausal.

History of a prior stroke is an intuitive factor affecting acute blood pressure control since the brain plays a key role in regulation of blood pressure. It has been shown that blood pressure rises in the first 24 hours after stroke to increase perfusion to the damaged area of the brain.^17^ It is also noted that blood pressure will trend down over the next week, and then plateau. Just as previous stroke deficits can be exacerbated during acute illness, it is possible that the brain may reset the autoregulation curve for blood pressure control during times of acute stress, making it more difficult for antihypertensives to be effective.

End organ damage will also intuitively lead to difficulty controlling blood pressure, as injury to the brain and kidneys affect the regulation of blood pressure. Persistence of elevated blood pressure leads to endothelial damage, promoting platelet aggregation and fibrin deposition, which stimulates release of further inflammatory molecules and perpetuates cycle of damage.^18^ Angiotensin II is thought to play a large role in the damage to organs associated with hypertension, so the medicines used in this study may have been ineffective in lowering blood pressure in the setting of end organ damage because they were targeting the wrong pathway.^18^ There is suggestion of kidney injury present in the non-responders, as they did have higher creatinine levels, despite no difference in rate of patients requiring dialysis between the responders and non-responders.

There are multiple studies to guide choice of IV antihypertensive in the setting of specific end organ damage such as stroke, MI, aortic dissection and end stage renal disease, but no recommendation for an IV antihypertensive that could be started based on suspicion alone of end organ damage.^19^ We chose to define end organ damage based on presenting symptoms because emergency physicians are frequently faced with the decision to treat critically elevated blood pressure before the results of diagnostic testing are available.

## LIMITATIONS

There are a number of limitations to our study. First, we used only two IV antihypertensives, so patients labeled as non-responders are not necessarily unresponsive to all IV antihypertensives. We chose a two medication study model because it allowed direct comparison of two of the most common classes of IV antihypertensives currently used in U.S. emergency departments and there is no gold standard medication for blood pressure control. The selected agents were relatively easy to titrate with a low side effect profile. However, there are differences in the method of administration between labetalol and nicardipine (bolus vs. infusion) that may account for our results, in that an infusion may inherently reach the target BP range faster than bolus therapy. Our study was not designed to determine the effects of administration method on time to BP control.

Secondly, the treating physicians had control over multiple variables. FDA recommendations are for titration of the nicardipine at intervals of 5 minutes, while the labetalol is re-dosed at 10 minute intervals. However, the actual titration intervals and dosing were left to the discretion of the treating physician, which may have biased the study. It is possible that more aggressive dosing of the medications might have altered results. Importantly, there were no differences in the number of times the two drugs were titrated, although there were more non-responders in the labetalol group. The treating physicians also set the goal blood pressure. Common recommendations for hypertensive emergency call for lowering the blood pressure by 25% over hours. However, there are individual patient characteristics that may affect the timing and degree of lowering. Allowing the treating physicians to set the blood pressure goal reflects real world practice.

Thirdly, as this was a randomized controlled trial, the groups were similar in terms of baseline characteristics. However, the treating physicians were not blinded to study drug and therefore bias may have been introduced with regard to the effect of allocated treatments. Due to the low number of patients that were defined as non-responders, there was a limited amount of covariates that could be analyzed and there may be further factors that are associated with nonresponse that we failed to identify.

Using systolic blood pressure alone may have failed to identify patients with isolated elevated diastolic blood pressure who may be at risk for hypertensive emergency. It is unclear if those patients would have a different response to medication therapy. It was acknowledged by JNC 7 that diastolic pressure levels out and may actually fall after the age of 50, making systolic blood pressure a more potent cardiovascular risk factor in patients over the age of 50.

Finally, CLUE only reported BP response within the first 30 minutes of antihypertensive use. It is unknown if those patients that were resistant within this time period continued to be resistant, or if it just took longer for them to respond. However, it can be argued that BP control requiring more than 30 minutes to attain is insufficient in the setting of hypertensive emergency. Labetalol has an onset of action of just five minutes and a peak effect is reached by thirty minutes. Nicardipine has an onset of action within 5–15 minutes, although the peak effect is not reached until about 45 minutes. This knowledge would suggest that a longer time period may be needed to assess non-response to nicardipine, but this study found more non-responders in the labetalol group.

## CONCLUSION

In this secondary analysis from the CLUE study, we have identified patient characteristics associated with difficulty in the emergency department control of BP within 30 minutes of the initiation of therapy. These characteristics include being male and having a previous history of stroke.

## Figures and Tables

**Table 1:** Characteristics of patients by responder status.

	Met target SBP within 30 minutes		
	
	Total (n=223)	No (n=29)	Yes (n=194)
Randomization cohort			
Nicardipine, n (%)	109 (48.9)	9 (31.0)	100 (51.5)
Labetalol, n (%)	114 (51.1)	20 (69.0)	94 (48.5)
Demographics			
Age, years, mean ± SD	52.4 ± 14.5	52.0 ± 15.2	52.5 ± 14.4
Female, n (%)	118 (52.9)	9 (31.0)	109 (56.2)
African American, n (%)	171 (77.0)	23 (79.3)	148 (76.7)
White, n (%)	50 (22.5)	6 (20.7)	44 (22.8)
Hispanic/Latino, n (%)	12 (5.4)	2 (6.9)	10 (5.2)
Social history			
Prior smoking, n (%)	130 (58.3)	16 (55.2)	114 (58.8)
Prior stimulant use, n (%)	40 (17.9)	8 (27.6)	32 (16.5)
Past medical history			
Hypertension, n (%)	211 (95.1)	27 (93.1)	184 (95.3)
Previous admission for hypertensive crisis, n (%)	77 (37.2)	13 (48.2)	64 (35.6)
Hyperlipidemia, n (%)	75 (35.1)	7 (25.9)	68 (36.4)
Diabetes, n (%)	62 (27.9)	6 (20.7)	56 (29.0)
Coronary artery disease, n (%)	30 (13.6)	2 (7.1)	28 (14.6)
Dialysis, n (%)	28 (12.7)	7 (24.1)	21 (10.9)
Stroke, n (%)	16 (7.3)	5 (17.9)	11 (5.8)
Heart failure, n (%)	20 (9.1)	2 (7.1)	18 (9.4)
Myocardial infarction, n (%)	13 (5.9)	3 (10.3)	10 (5.2)
Baseline lab/ECG			
Creatinine, mg/dL, median (IQR)	1.2 (0.9, 2.4)	1.8 (1.0, 7.6)	1.1 (0.8, 2.0)
BNP, pg/dL, median (IQR)	346 (131, 2184)	1466 (520, 2184)	244 (131, 905)
Troponin I, ng/mL, mean ± SD	0.2 ± 0.9	0.3 ± 0.9	0.2 ± 0.9
Abnormal ECG, n (%)	52 (28.0)	8 (30.8)	44 (27.5)

*SBP*, systolic blood pressure; *ECG,* electrocardiogram*; BNP*, B-type natriuretic peptide

**Table 2:** Univariate analysis of factors associated with nonresponse to intravenous antihypertensives.

	Responders to therapy within 30 minutes		
	
Parameter	No (n=29)	Yes (n=194)	P-Value
Nicardipine treatment	9 (31%)	100 (51.5%)	
Labetalol treatment	20 (69%)	94 (48.5%)	
Female	9 (31%)	109 (56%)	0.011
Altered level of consciousness	4 (13.8%)	2 (1%)	0.003
Epigastric pain	7 (24.1%)	17 (8.8%)	0.022
Palpitations	4 (13.8%)	6 (3.1%)	0.028
Dialysis dependent	7 (24.1%)	21 (10.9%)	0.067
Prior stroke	5 (17.9%)	11 (5.8%)	0.038
Prior antiadrenergic use	6 (20.9%)	15 (7.7%)	0.038
Creatinine (mg/dL)	4.1 (± 5.0)	2.3 (± 2.9)	0.026

**Table 3:** Hemodynamic response to treatment in responders versus non-responders.

	Met target SBP within 30 minutes			
	
Parameter	Total (N=223)	No (N=29)	Yes (N=194)	p-value
Number of titrations, n (%)*				0.412
0	33 (14.8)	2 (6.9)	31 (16)	
1	62 (27.8)	7 (24.1)	55 (28.4)	
2	49 (22)	9 (31)	40 (20.6)	
>2	79 (35.4)	11 (37.9)	68 (35.1)	
Number of instances within TSBP range (Mean ± SD)	3.5 ± 2.0	0.0 ± 0.0	4.0 ± 1.6	<0.001
Total nicardipine dose (Mean ± SD) mg	3.6 ±1.5	5.2 ±2.0 (n=9)	3.4 ± 1.4 (n=100)	0.012
Total labetalol dose (Mean ± SD) mg	58.2 ± 39.2	77.0 ±39.6 (n=20)	54.1 ± 38.2 (n=94)	0.006
Patient SBP above target, n (%)	199 (89.2)	28 (96.6)	171 (88.1)	0.330
Patient SBP below target, n (%)	27 (12.1)	1 (3.4)	26 (13.4)	0.217
HR below 60, n (%)	23 (10.9)	4 (14.8)	19 (10.3)	0.507

*SBP*, systolic blood pressure; *TSBP,* target systolic blood pressure; *HR,* heart rate

* Number of titrations indicates number of doses for labetalol and number of titrations of the drip for nicardepine.

**Table 4:** Distribution of non-responders by distance outside target range.

	Number (%) of non-responders
0–5mmHg outside range	11 (37.9)
6–10mmHg outside range	5 (17.2)
11–15mmHg outside range	6 (20.7)
16–20mmHg outside range	3 (10.3)
>20mmHg outside range	4 (13.8)
